# *Chryseobacterium Indologenes* Sepsis and Acute Renal Failure Secondary to Abdominal Compartment Syndrome in a Confirmed COVID-19 Patient

**DOI:** 10.1155/2022/7946158

**Published:** 2022-04-14

**Authors:** Alejandro José Quiroz Alfaro, Iván Javier Rodríguez Acosta, Mayumi Tanaka Takegami, Liliana Michelle Bracho Maya, Roberto Eduardo Quiroz Simanca

**Affiliations:** ^1^Universidad Colegio Mayor de Nuestra Señora del Rosario, Bogotá, Colombia; ^2^Unidad de Cuidados Intensivos, Clínica Erasmo, Valledupar, Cesar, Colombia

## Abstract

Sepsis due to nosocomial pathogens markedly increases morbidity and mortality in the critically ill patient. The SARS-CoV-2 (COVID-19) pandemic has increased the number of patients requiring intensive care unit (ICU) in-patient management. *Chryseobacterium indologenes* (*C. indologenes*) is a group of multiresistant gram-negative bacteria associated with in-hospital environment and catheter-associated infections of increasing importance in the ICU. SARS-CoV-2 severe infection in the critically ill patient increases the risk of abdominal compartment syndrome (ACS) and acute kidney injury (AKI). We hereby report a case of a patient with SARS-CoV-2 severe infection, *C. indologenes* sepsis, abdominal compartment syndrome, and secondary renal failure.

## 1. Introduction

SARS-CoV-2 is a novel coronavirus of coronaviridae family. It is initially documented in Wuhan, China, causing acute respiratory infection, pneumonia, sepsis, and acute respiratory distress syndrome (ARDS), amongst other complications. Due to the increase in COVID-19 cases on March 11, 2020, a pandemic was declared, becoming a challenge for the management of the critically ill patient [1].

COVID-19 is a positive-strand, enveloped, RNA virus, that infects its host cells via the angiotensin-converting enzyme 2 receptor (ACE-2) using the spike protein (S protein) as a binding ligand [1].

Although COVID-19 mainly affects the respiratory tract, extrapulmonary involvement is also common. AKI is present in up to 40% of in-hospital COVID-19 infected patients negatively impacting patient survival [2].

Hospital-acquired pneumonia and ventilator-associated pneumonia are two potentially fatal septicemic entities in the critically ill patient [3].

*C. indologenes* is a nonmotile gram-negative, catalase-positive, oxidase-positive, indole-positive, and nonfermenting bacillus rarely found in the human bacterial microflora, although widely distributed in nature. *C. indologenes* can cause bacteremia, pneumonia, sepsis, and meningitis with an increased incidence on critically ill and immunocompromised patients [4, 5].

*C. indologenes* is a multiresistant nosocomial bacteria uncommonly found as a cause of bacteremia; the absence of treatment guidelines or expert consensus makes the treatment challenging [5].

ACS is commonly found on critically ill patients and has cardiovascular, pulmonary, and renal deleterious effects [6].

COVID-19, nosocomial infections, and ACS have become entities of great importance in the critically ill patient. In our experience, the coexistence of any of these pathologies becomes a therapeutic challenge for the critical care physician. We consider that our case highlights the importance of suspecting ACS in the context of AKI in the critically ill patient.

## 2. Case

**Figure 1:**
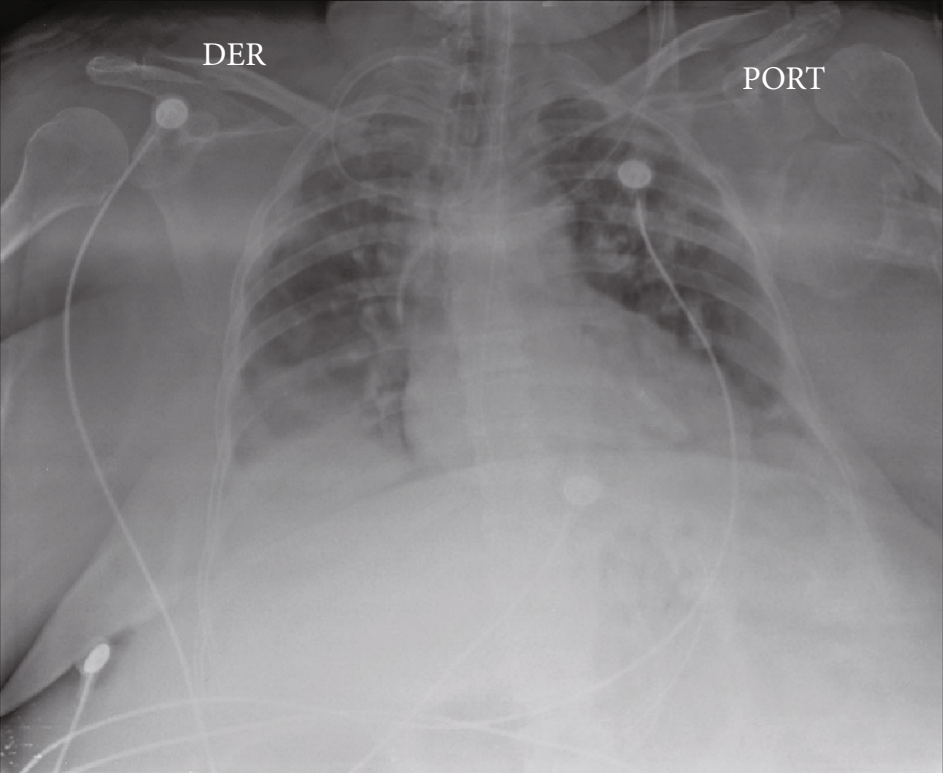
Portable chest X-ray ray revealing bilateral interstitial and alveolar infiltrates with right lung basal consolidation.

A 70-year-old female patient with a history of obesity and hypertension presented to the emergency room with severe dyspnea, fever, headache, cough, and colicky abdominal pain. Physical examination was remarkable for temperature of 38.3°C (101°F), blood pressure 122/86 mmHg, heart rate 112 beats per minute, respiratory rate 32 breaths per minute, oxygen saturation 88%, intercostal and supraclavicular retractions, generalized crackles on both lungs, and generalized abdominal tenderness with no signs of peritoneal irritation. Portable chest X-ray revealed bilateral interstitial and alveolar infiltrates with right lung basal consolidation (Figure 1). Initial work-up revealed an elevated blood cell count of 11,700/*μ*l with neutrophils predominance (91%), hemoglobin 10.8 g/dL, PTT 38 seconds, D-dimer 973 mcg/mL, C-reactive protein 22.7 mg/dL, creatinine 0.87 mg/dL, BUN 18.7 mg/dL, PCR for SARS-CoV-2 positive, and arterial blood gas (ABG) showing respiratory acidosis with a PaO_2_/FiO_2_ of 200. Antiviral therapy and broad-spectrum antibiotics with Sulbactam/ampicillin and tocilizumab were started for COVID-19 and empiric coverage for community-acquired pneumonia. Initial diagnosis of ARDS was made, and the patient was intubated and translated to the intensive care unit (ICU).

She persisted clinically stable with relative improvement on her oxygenation and ventilation patterns; however, hemoglobin levels continued diminishing up to 7.2 g/dL requiring a blood transfusion. Nasogastric lavage returned clear liquid, occult blood on stool was negative, and no other evidence of gastrointestinal bleeding were found. Endotracheal tube, urine culture, and blood cultures were taken to optimize and direct medical treatment.

**Figure 2:**
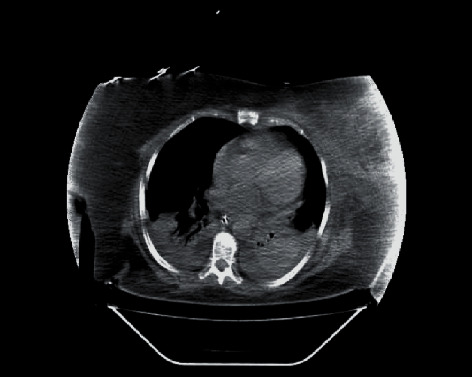
Chest tomography showing bilateral pleural effusion, bilateral basal subsegmental atelectasis, and generalized ground glass opacifications.

On her sixth day in the ICU, endotracheal tube and blood cultures grew *C. indologenes* multisensitive, and antibiotic therapy was switched to trimethoprim sulfamethoxazole following the indications of the infectious disease specialist. Chest tomography showed bilateral pleural effusion and bilateral basal subsegmental atelectasis associated with generalized ground glass opacifications (Figure 2), and after several days of gradual creatinine elevations and persistent fluid overload (positive fluid balance of 4.7 liters), despite adequate treatment for severe sepsis, fluid, and nutritional optimization, acute kidney injury stage 3 was established; nephrology was consulted and continuous venovenous hemodialysis was started.

Ventilatory pattern deteriorated and marked hypotension, with persistent inflammatory response in laboratory tests, so a diagnosis of septic shock was made, vasopressor support with norepinephrine was started, and antibiotic therapy was switched to vancomycin and meropenem.

**Figure 3:**
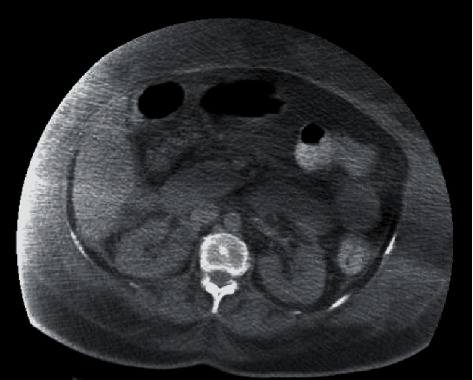
Abdominal tomography evidencing marked increase of the abdominal wall soft tissues' density.

During routine physical examination, abdominal examination revealed marked abdominal distention with increased abdominal girth. Swelling and induration of the left thigh were found, and Doppler ultrasound revealed common femoral vein thrombosis. Anticoagulation was initiated. Intraabdominal pressure was measured reporting 23–25 mmHg and abdominal tomography evidenced marked increase of abdominal wall soft tissues density (Figure 3). Liver failure, pancreatitis, and hypoalbuminemia were ruled out. ACS was diagnosed, secondary to AKI and fluid overload causing an abdominal restrictive process. Despite optimal medical treatment, the patient continued deteriorating and surgical approach was suggested; however, the patient's husband declined all possible surgical interventions so medical therapy continued.

Despite medical treatment, the patient continues with progressive clinical deterioration, persistent hypotension despite high doses of double vasopressor support with norepinephrine and vasopressin. Laboratory tests showing ABG with a severe metabolic acidosis and PaO_2_/FiO_2_ of 115 with FiO2 of 95% and PEEP of 14, BUN 40 mg/dL, creatinine 1.75 mg/dL, and hyperkalemia of 6.6 mEq/L, ventilatory support, broad-spectrum antibiotic therapy, and continuous hemofiltration due to renal failure, uremia, and hemodynamical instability. The patient develops cardiac arrest and unfortunately dies.

## 3. Discussion

SARS-CoV-2 is a virus with high affinity for the respiratory epithelium due to the high presence of ACE-2 receptors: its target receptor for entry into the host cell in the pulmonary parenchyma [1]. Initially, it may present an asymptomatic phase, with an average incubation period of up to 14 days; afterwards, it can continue an asymptomatic course or present with mild, moderate, or severe symptoms [1].

COVID-19 has person-to-person transmission. The most common diagnostic methods are real-time polymerase chain reaction and serological tests with sensitivities and specificities greater than 95% [1]. Treatment varies depending on the severity of the symptoms, starting with symptomatic treatment in mild cases up to oxygen therapy, corticosteroids, and antivirals in severe cases [1].

The incidence of nosocomial infections on critically ill patients with COVID-19 in the ICU has been reported up to 37% [7]. Nosocomial infections are considered a late complication COVID-19 infection on the critically ill patient in the ICU, associated with a prolonged in-hospital stay and responsible for the death in up to 33% of the critically ill patients with COVID-19 in the ICU [7].

Corticosteroids, tocilizumab, and prolonged hospital stay are risk factors for the development of coinfections on critically ill patients with COVID-19 in the ICU [7].

Hypertension, diabetes, chronic kidney disease, advanced age, and immunosuppression are risk factors for *C. indologenes* infection and bacteremia [5].

Optimal treatment for *C. indologenes* bacteremia is not standardized because of the lack of specific treatment guidelines. However, *C. indologenes* is usually sensitive to fluoroquinolones, piperacillin-tazobactam, ceftazidime, and cefepime [8]. Trimethoprim-sulfamethoxazole has shown in vitro activity against *C. indologenes* in up to 60% of isolates in vitro studies [8].

*C. indologenes* isolates on in vitro studies have intrinsic resistance to aztreonam, aminoglycosides, aminopenicillins, and first-generation cephalosporins and are sensitive to quinolones and trimethoprim sulfamethoxazole in up to 95% of cases, with an average treatment duration of 7–14 days [9].

Treatment of AKI associated with COVID-19 infection is optimized to avoiding nephrotoxic medications, strict monitoring of ABG, fluid balance, creatinine, urine output, and hemodynamic monitoring, considering renal replacement therapy and extracorporeal support in patients with significant renal injury who meet the necessary criteria [10].

AH and ACS are two entities to be considered in patients with acute renal failure who do not improve despite adequate medical management like we presented in our case.

IAP can be measured instillating intravesical saline solution, a safe and minimally invasive method [11]. Decreased abdominal wall compliance, intraabdominal collections, increased intraluminal contents, and large intravenous fluid volumes during resuscitation are risk factors for AH [11].

Normal intraabdominal pressure is 5-7 mmHg. Abdominal hypertension (AH) is defined as any intraabdominal pressure (IAP) greater than 12 mmHg, and abdominal compartment syndrome (ACS) is defined as a pressure that is maintained above 20 mmHg and may be associated with some secondary organ or multisystemic alteration [11].

IAP ≥ 10 mmHg can generate a significant decrease in urine output due to decreased renal perfusion and increased vascular resistance [12], possibly one of the many factors that exacerbated the AKI in our case.

ACS treatment is aimed at improving abdominal compliance, evacuating abdominal collections and intraluminal contents, correcting positive fluid balance, and giving hemodynamic support if required [12].

Indications for surgical treatment are failure of medical management and rapidly progressive organ dysfunction caused by AH. Early abdominal closure is always preferred [13].

Laparostomy and open abdomen, if indicated, are possible surgical treatment options. These procedures may be associated with complications such as nutritional alterations, protein and fluid loss, loss of abdominal domain, frozen abdomen, and enteroatmospheric fistulas [14].

There is evidence that suggests that in a dysfunctional immune system, a cytokine storm can be produced as a late inflammatory response secondary to the COVID-19 infection that significantly deteriorates the patient's condition, resulting in a hyperinflammatory state, exacerbating multiorgan failure [15], features observed in our patient that most likely worsened the septic shock and hindered the clinical outcome.

Our case emphasizes the importance of the rapid diagnostic and treatment of patients with COVID-19 and its complications like nosocomial infections in the critically ill patient and the rapid initiation of broad-spectrum antibiotics and adequate pathogen-directed therapy once bacterial resistance is known. It is important to suspect infections with atypical nosocomial pathogens like *C. indologenes* since the multiresistance of some nosocomial bacteria can worsen clinical outcomes and delay adequate antimicrobial treatment, negatively impacting patient survival. The treatment of AKI in the critically ill patient in the ICU setting can improve clinical outcomes; however, ACS should be suspected as cause or exacerbating factor in patients that do not improve with initial medical therapy, as in our case. Despite optimal treatment, the critically ill patient remains a diagnostic and therapeutic challenge.

## 4. Conclusions

SARS-CoV-2 infection may present with severe disease requiring multidisciplinary in-patient management in the ICU. SARS-CoV-2 infection in the critically ill patient increases the risk of complications such as AKI and abdominal compartment syndrome. AH and ACS should be suspected in critically ill patients with poor clinical evolution, especially in the context of acute kidney injury. Medical management of ACS remains the mainstay of treatment although surgical treatment remains a plausible option in some cases.

## Data Availability

All the information on this case report was obtained from the patient, close family members, and medical records with appropriate consent.

## Abbreviations

SARS-CoV-2: COVID-19
ICU: intensive care unit
*C. indologenes*: *Chryseobacterium indologenes*
ACS: abdominal compartment syndrome
AKI: acute kidney injury
ARDS: acute respiratory distress syndrome
ACE-2: angiotensin-converting enzyme 2 receptor
ABG: arterial blood gas
AH: abdominal hypertension
IAP: intraabdominal pressure
